# Deprescribing to reduce polypharmacy: study protocol for a randomised controlled trial assessing deprescribing of anticholinergic and sedative drugs in a cohort of frail older people living in the community

**DOI:** 10.1186/s13063-021-05711-w

**Published:** 2021-11-03

**Authors:** Ulrich Bergler, Nagham J. Ailabouni, John W. Pickering, Sarah N. Hilmer, Dee Mangin, Prasad S. Nishtala, Hamish Jamieson, Sponsor-investigator

**Affiliations:** 1 Department of Medicine, University of Otago, Christchurch, New Zealand; 2 UniSA Clinical & Health Sciences, Quality Use of Medicines and Pharmacy Research Centre, University of South Australia, Adelaide, South Australia; 3 Geriatric Pharmacology, Faculty of Medicine and Health, Northern Clinical School, Kolling Institute, University of Sydney and Royal North Shore Hospital, St Leonards, Australia; 4 David Braley and Nancy Gordon Chair in Family Medicine, McMaster University, Hamilton, Canada; 5 Department of Pharmacy & Pharmacology, University of Bath, Bath, UK; 6 Burwood Hospital, Canterbury District Health Board, Christchurch, New Zealand; 7 Health Research Council of New Zealand, Level 3/110 Stanley Street, Grafton, Auckland, 1010 New Zealand

**Keywords:** Deprescribing, Elderly, Polypharmacy, Anticholinergic, Sedatives, Drug burden index, interRAI

## Abstract

**Background:**

Targeted deprescribing of anticholinergic and sedative medications in older people may improve their health outcomes. This trial will determine if pharmacist-led reviews lead to general practitioners deprescribing anticholinergic and sedative medications in older people living in the community.

**Methods and analysis:**

The standard protocol items: Recommendations for Interventional Trials (SPIRIT) checklist was used to develop and report the protocol. The trial will involve older adults stratified by frailty (low, medium, and high). This will be a pragmatic two-arm randomized controlled trial to test general practitioner uptake of pharmacist recommendations to deprescribe anticholinergic and sedative medications that are causing adverse side effects in patients.

**Study population:**

Community-dwelling frail adults, 65 years or older, living in the Canterbury region of New Zealand, seeking publicly funded home support services or admission to aged residential care and taking at least one anticholinergic or sedative medication regularly.

**Intervention:**

New Zealand registered pharmacists using peer-reviewed deprescribing guidelines will visit participants at home in the community, review their medications, and recommend anticholinergic and sedative medications that could be deprescribed to the participant’s general practitioner. The total use of anticholinergic and sedative medications will be quantified using the Drug Burden Index (DBI).

**Outcomes:**

The primary outcome will be the change in total DBI between baseline and 6-month follow-up. Secondary outcomes will include entry into aged residential care, prolonged hospitalization, and death.

**Data collection points:**

Data will be collected at the time of interRAI assessments (T0), at the time of the baseline review (T1), at 6 months following the baseline review (T2), and at the end of the study period, or end of study participation for participants admitted into aged residential care, or who died (T3).

**Ethics and dissemination:**

Ethical approval has been obtained from the Human, Disability and Ethics Committee: ethical number (17CEN265).

**Trial registration:**

ClinicalTrials.gov ACTRN12618000729224. Registered on May 2, 2018, with the Australian New Zealand Clinical Trials Registry

**Supplementary Information:**

The online version contains supplementary material available at 10.1186/s13063-021-05711-w.

## Background

Polypharmacy, the use of five or more of medications, is prevalent amongst older adults positioning them at a higher risk of drug-drug interactions and one or more inappropriate medications. Inappropriate medications are defined as those whose potential harm outweighs their possible benefits currently in the individual [1]. Deprescribing, the process of safely reducing or discontinuing unnecessary or harmful medicines with clinical supervision, can decrease polypharmacy, reduce inappropriate medicine use, and may improve health outcomes [2].

Disease-specific guidelines offer little advice regarding deprescribing potentially harmful medications [3, 4] for people with multimorbidity. Of note, anticholinergic and sedative medications can be inappropriately prescribed for older adults [5]. The cumulative, long-term use of these medications is associated with several negative health consequences, including impaired muscle strength, worsening cognition, increased frailty, poorer physical functioning (e.g., balance), heightened risk of falls, increased rate of hospitalizations, entry into residential care, and even death [6, 7]. Polypharmacy is associated with frailty [8], a geriatric syndrome present in many older adults. Frailty increases with age due to age-related physiological deterioration. Up to half of the people aged over 85 years experience frailty [2]. Small trigger events, like a seasonal illness or fall, can cause a sudden decline in health and negative outcomes in already frail individuals [2, 9, 10]. Little is known on how a quantified measure of frailty could inform and help in targeting frail individuals for deprescribing.

Deprescribing of unnecessary anticholinergic and sedative medications has been shown to potentially improve health outcomes of older people [2, 9], including cognition [10], and reducing the risk of falls [11] and hip fractures. In addition, although inconclusive to date, evidence suggests that deprescribing could improve reported quality of life [12, 13].

Although previous studies have demonstrated the feasibility and overall safety of deprescribing, questions remain regarding how best to do it in practice [14, 15]. In a multi-disciplinary residential aged care setting, pharmacist-led interventions have successfully reduced unnecessary prescribing of sedative and anticholinergic medications [16, 17]. A cluster randomized controlled trial of clinical decision support software targeting deprescribing anticholinergic and sedative medications for pharmacists conducting the Home Medicines Review in Australia increased pharmacists’ recommendations to reduce anticholinergic and sedative medications but not their uptake [18].

The drug burden index (DBI) is a linear, additive pharmacological model that uses pharmacokinetic and pharmacodynamic principles to calculate an individual’s total exposure to anticholinergic and sedative medications [14]. Exposure to each additional unit of DBI has been shown to have a negative effect on the physical function of older people, equivalent to three additional physical comorbidities [14]. An association between increasing DBI and impaired function has been demonstrated in a cross-sectional analysis of two populations of older people in the USA [19], in older Australian men [20], and a longitudinal study of community-dwelling older people in the USA [21]. Furthermore, increasing DBI is associated with prevalent and incident frailty [22] and frailty transitions [23].

In this randomized controlled trial in a community setting, we will test a targeted approach to pharmacist-led deprescribing of medications contributing to DBI in frail older adults. Specifically, this study will test the utility of a frailty measure for targeting deprescribing on individuals with the greatest potential for improvement. We will identify older adults seeking funded care support who undergo a structured, comprehensive need assessment, develop a frailty measure using data obtained through these assessments, and stratify our participants into three groups based on their frailty index. Pharmacists will visit participants in their homes, record current medication use, and develop recommendations for deprescribing, which are passed to the patient’s primary physician for consideration. We will measure changes in DBI medication use and differences in hospitalization and entry into aged residential care.

## Methods

### Hypotheses and aims

We hypothesize that implementing a pharmacist-led medication review and provision of deprescribing recommendations to general practitioners (GP) will reduce the use of anticholinergic and sedative medications in community-dwelling older people compared to the control arm overall and that the reduction will be more pronounced for older people with a greater level of frailty. Thus, the primary objective is to determine if pharmacist-led medication reviews focused on reducing anticholinergic and sedative medications lead to GPs deprescribing these medications for older people living in the community. The secondary objective is to determine if a frailty measure based on data collected in the interRAI Home Care [24] and Contact assessment [25] can identify a group of older people who could benefit the most from deprescribing.

The interRAI is a comprehensive assessment database system utilized internationally and in New Zealand to standardize the evaluation of complex care needs of older people. It is routinely used to collect data regarding patients’ medical and functional status [26]. In New Zealand, the comprehensive Home Care assessment [24] is used for persons seeking admission into publicly funded aged residential care, while the shorter contact assessment [25] is for home-based support services. The reliability of the interRAI™ has been tested, and it has been shown to meet or exceed the standard cut-offs for acceptable reliability [27].

### Study design

This will be a pragmatic two-arm randomized controlled superiority trial to test general practitioner uptake of pharmacist recommendations to deprescribe anticholinergic and sedative medications that are causing adverse side effects in patients. The cumulative use of anticholinergic and sedative medications listed in Table 1 for each participant will be quantified using the DBI [14]. New Zealand registered pharmacists will use previously pilot-tested deprescribing guidelines to recommend to GPs sedative and anticholinergic medications with a potential to be deprescribed [28, 29].

**Table 1:** Anticholinergic and sedative medications

Generic medicine name	ATC code	Generic medicine name	ATC code
Alprazolam	N05BA12	Methyldopa	C02AB01
Amitriptyline	N06AA09	Metoclopramide	A03FA01
Aripiprazole	N05AX12	Mianserin	N06AX03
Amantadine	N04BB01	Mirtazapine	N06AX11
Benztropine	NO4AC01	Moclobemide	N06AG02
Benzhexol	N04AA01	Morphine	NO2AA01
Biperidin	N04AA02	Nitrazepam	N05CD02
Buprenorphine	N02AE01	Nortryptyline	N06AA10
Buspirone	N05BE01	Olanzapine	N05AH03
Carbamazepine	N03AF01	Orphenadrine	N04AB02
Cetirizine	R06AE07	Oxazepam	N05BA04
Chlorpheniramine	R06AB05	Oxybutynin	G04BD04
Chlorpromazine	N05AA01	Oxycodone	N02AA05
Citalopram	N06AB04	Paroxetine	N06AB05
Clemastine	D04AA14	Pericyazine	NO5AC01
Clomipramine	N06AA04	Phenobarbital	N03AA02
Clonazepam	N03AE01	Phenytoin	N03AB02
Clonidine	S01EA04	Pizotifen	N02CX01
Codeine	R05DA04	Pramipexole	N04BC05
Cyproheptadine	R06AX02	Prazosin	C02CA01
Darifenacin	G04BD10	Pregabalin	N03AX16
Dexchlorpheniramine	R06AB02	Primidone	N03AA03
Dextromethorphan	N02AC04	Prochlorperazine	N05AB04
Diazepam	N05BA01	Promethazine	R06AD02
Dihydrocodeine	N02AA08	Quetiapine	NO5AH04
Diphenhydramine	D04AA32	Risperidone	N05AX08
Disopyramide	C01BA03	Ropinirole	N04BC04
Doxazosin	C02CA04	Selegiline	N04BD01
Dothiepin	N06AA16	Sertraline	N06AB06
Doxepin	N06AA12	Solifenacin	G04BD08
Escitalopram	N06AB10	Tamsulosin	G04CA02
Fentanyl	N02AB03	Temazepam	N05CD07
Fexofenadine	R06AX26	Terazosin	G04CA03
Flunitrazepam	N05CD03	Tolterodine	G04BD07
Fluoxetine	N06AB03	Tramadol	NO2AX02
Fluphenazine	NO5AB02	Tranylcypromine	N06AF04
Fluvoxamine	N06AB08	Triazolam	N05CD05
Gabapentin	N03AX12	Trifluoperazine	N05AB06
Haloperidol	N05AD01	Trihexyphenidyl	N04AA01
Imipramine	N06AA02	Trimipramine	N06AA06
Lamotrigine	N03AX09	Valproic Acid	N03AG01
Levetiracetam	N03AX14	Venlafaxine	N06AX16
Loperamide	A07DA03	Ziprasidone	N05AE04
Loratadine	R06AX13	Zopiclone	N05CF01
Lorazepam	N05BA06	Zolpidem	N05CF02
Lormetazepam	N05CD06	Zuclopenthixol	N05AF05
Methadone	N07BC02		

After enrolment, participants will be stratified using a frailty index (FI), which will be based on a cumulative deficit model using 15 relevant items common to the interRAI Homecare and Contact assessments. Cut-off values for each stratum will create three equal-sized frailty strata based on New Zealand interRAI HC and CA data used for developing the frailty index. Participants will be stratified into one of three frailty strata (low, medium, high) and allocated in a 1:1 ratio within each strata to the intervention and control arms of the trial.

### Study setting

This trial will be conducted at the University of Otago, Christchurch, in the Canterbury provincial region of New Zealand. Study pharmacists will visit participants in their homes, where they will review medication use at the time of their visit.

### Participants

Participants will be eligible for inclusion if they are as follows:

1. Are aged ≥65 years
2. Have undergone an interRAI Home Care [24] or contact [25] assessment
3. Are regularly taking at least one anticholinergic or sedative medication as shown in the interRAI or dispensing records.

Participants will be excluded from the study for any of the following reasons:

1. Not consenting for their interRAI data to be used for research
2. Having a psychiatric disorder or dementia disease (e.g., Alzheimer’s disease, dementia, schizophrenia, abnormal thought processes, delusions, hallucinations)
3. Scoring 3 or higher on the interRAI Cognitive Performance Scale
4. Having a terminal illness with life expectancy ≤6 months
5. Determined as non-frail by having no deficits in the frailty index
6. Having an initial DBI score of <0.5 (DBI score of 0.5 is the equivalent of one DBI medication being taken at the minimum efficacious dose) [14].
7. Having a potentially life-threatening drug interaction requiring urgent medical intervention (during the study period)

### Procedure

**Fig. 1:**
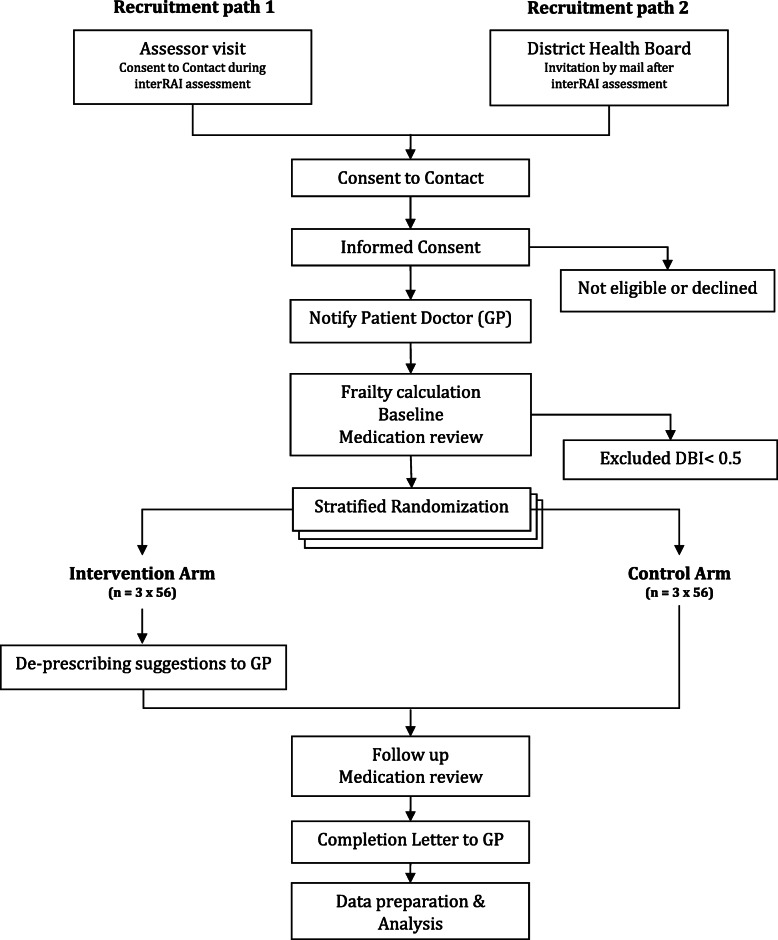
Study process flowchart

The study process flow is depicted in Fig. 1—“study process flow chart” and each step detailed in the following section of this protocol.

#### Recruitment

There will be three layers of consent involved in this study: (1) when people undergo an interRAI assessment, they are asked to consent for their data to be used for planning and research; (2) when the assessing health care provider asks the person to give consent to be contacted by the research team; and (3) the person consents to take part in the trial.

Potential participants will be contacted via two pathways: (1) interRAI assessors will invite eligible older adults to participate in the study during their interRAI assessment home visit and (2) the local district health board will post letters to older adults who have recently had an interRAI assessment. The letters will outline a brief overview of the study with “Consent to Contact” (CtC) forms and free post return envelopes.

Once the research team has received the signed CtC forms, the study administrator will contact potential participants and explain the study in detail. The CtC form has two tick boxes whereby a potential participant can (1) permit the study team to contact their community pharmacist to confirm regular prescriptions of sedative or anticholinergic medications and (2) permit access to the electronic interRAI assessment record. This information will confirm the medication participants are taking at the start of the study.

The study administrator will confirm prescribed medications with participants during enrolment and their eligibility to participate in the study. If potential participants indicate verbally their willingness to take part in the study, a home visit by a study pharmacist will be scheduled. During visits, study pharmacists will explain the study to the potential participants, answer any questions, and obtain informed written consent to take part in the study.

Once consent to participate in the study has been received, each participant’s GP will be advised in writing that their patient has consented to take part in this study. Participants may withdraw from the study at any time.

#### Stratification

Using each consenting participant’s interRAI data, the Study Administrator will calculate their frailty index (FI) and classify each participant into one of the three frailty strata: low, medium, or high before the pharmacist’s visit. Details for the stratification methods can be found in the paragraph outlining the study design.

#### Randomization and allocation

Predefined randomization lists with a 1:1 allocation will be calculated by the study’s data manager using a Mersenne Twister algorithm. The algorithm will be repeatedly run until a list is identified that shows a difference in the total number of intervention and control arm participants of not more than three participants at any given point in the process. Centralized allocation is considered neither viable nor required in this community-based study setting, as the participants become available at random through the interRAI assessment process. The randomization list is securely stored on the university’s computer system and not available to any other research team member. Each frailty strata will have its randomization list and set of treatment allocations concealed in sequentially numbered sealed opaque envelopes. Allocations will be made by the study administrator, who will select the next allocation matching a participant’s frailty strata when the participant’s home visit is booked. The envelopes are opened after completion of the pre-intervention medication review. If a participant is discovered to be ineligible during the pre-intervention medication review, the corresponding envelope is returned and used with the next available participant.

#### Baseline medication review

Participants' medication use will be reviewed before the intervention and at least 6 months following the intervention. However, to avoid unnecessary complexity in obtaining prescribing records for people living in community settings, the focus will be given to the in-person review process in the participants’ homes. Here, the pharmacist will inquire about, view, and record the daily dose of all the participant’s medications.

#### Intervention arm

In the intervention arm of the trial, pharmacists will discuss with participants the medical conditions with which the DBI medications have been prescribed and their experiences with these medications. The consultation will determine if the potential harm of DBI medications prescribed outweigh their possible benefits. The participant and their family’s beliefs about the continued need for these medications and their preferences regarding willingness to discontinue these medications will also be determined via the consultation. After the consultation, target anticholinergic/sedative medications will be documented along with the participant’s concerns regarding these medications. Study pharmacists will consider the participants’ medical history as conveyed by the participants, their interRAI assessments, and their electronic medication records, if available.

The deprescribing implementation strategy that will be employed will focus on reducing or stopping anticholinergic and sedative medications to reduce participants’ DBI scores, using a process that was adapted from [2] and previously trialled in a New Zealand residential aged care setting [28, 29]. The process used in this study will differ from that used in the above residential care study because our study pharmacists will not have access to prescribing lists and clinical records before meeting the participants. Nevertheless, face to face consultations with the study pharmacists will capture rich data on participants’ medical history, medication lists, and their beliefs about their medications.

The deprescribing medication review will involve the following:

- Pharmacist review of medication use
- Participant consultation
- Pharmacists suggesting changes in DBI medications to GPs
- GP consultation with the participants
- GP decision to revise or not to revise DBI medication prescriptions

All medication reviews and DBI calculations will be reviewed and verified independently by a senior pharmacist (author PSN). All suggestions developed on potential deprescribing will be peer-reviewed by another study pharmacist before sending them to the participant’s GP, with additional expertise being available from the senior pharmacist.

Table 1 lists all the target medications considered for deprescribing in this trial, along with their corresponding anatomical therapeutic classification (ATC) code [30]. These medications are classified as anticholinergic or sedative based on The New Zealand Formulary [31]. This group of medications will encompass antipsychotics, anti-depressants, and benzodiazepines and non-benzodiazepine hypnotics.

#### Control arm

If allocated to the control arm, the pharmacist will conduct the medication review without discussing deprescribing options with participants. The control group participants will continue to receive routine care from their GP.

#### Clinical responsibility

Clinical responsibility for all participants remains with their primary care GP. Participants will be asked to contact their GP if they feel unwell during drug withdrawal. If disease relapse occurs or unwanted adverse drug effects occur, the medicine will be re-prescribed as seen appropriate by a participant’s GP.

Reasons for withdrawal or dropout, other than death, will not be recorded in the study. However, the research team will access and analyze hospital admissions. In addition, any notification of incidents received from participants or their GPs will be passed onto an internal data monitoring committee for review (data monitoring and safety are explained in the latter part of the document).

#### Six months of follow-up

For all participants, the medication use will be recorded before randomization and repeated at least 6 months later. At a 6-month follow-up, research pharmacists will revisit participants and ascertain medication usage in both study arms using the same processes as the baseline review. To avoid bias and achieve blinding of the visiting pharmacist, each participant will be visited by a different pharmacist at follow-up than the one who saw them at baseline. The post-intervention medication review will be provided to the participants’ GP for information purposes only.

### Data management

#### Data collection methods

All observations and results will be recorded in custom-designed Research Electronic Data Capture (REDCap https://www.project-redcap.org/) databases hosted at the University of Otago, Christchurch, New Zealand, which will allow for secure online data entry from multiple sites into a central data repository [30, 31].

A REDCap database holding details on potential study participants will contain identifying personal data separate from the database holding de-identified data for consented study participants. Access to these databases is defined in the section “Access to data.”

Data will be collected at the time of the interRAI assessment (T0), during the preparation of the intervention (T1), at least 6 months following the baseline pharmacist’s review (T2), and at the end of the study period or end of study participation for admission into aged residential care or death (T3) as detailed in Table 2.

**Table 2:**
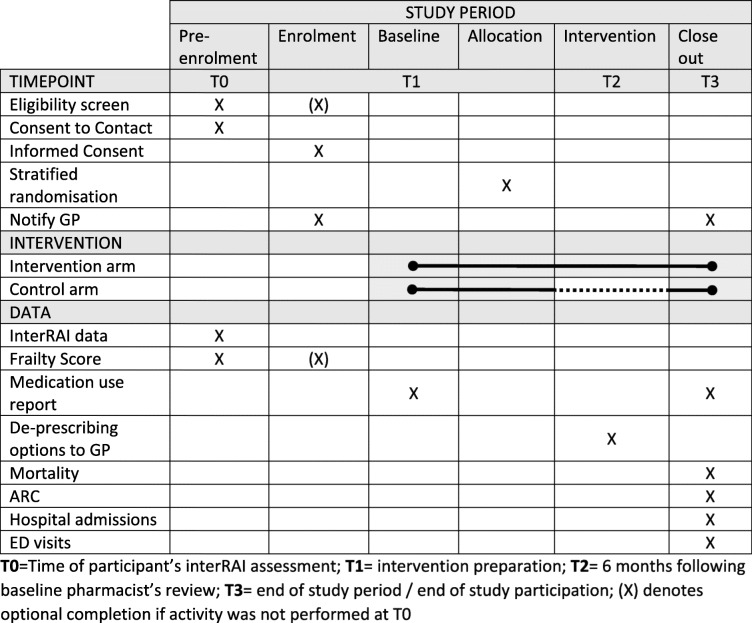
Participant data to be collected during the study

*T0* time of participant’s interRAI assessment, *T1* intervention preparation, *T2* 6 months following baseline pharmacist’s review, *T3* end of study period/end of study participation, *(X)* denotes optional completion if activity was not performed at T0

The medication use will be recorded before randomization and repeated at 6 months of follow-up. Secondary health information such as mortality, hospital admissions, and fractures will be obtained from the health provider and the New Zealand Ministry of Health. These data will be linked with the study data using each participant’s unique National Health Index (NHI) number [32].

No biological samples will be collected in this trial.

#### Data entry and quality checks

The pharmacist will record the medication review details on paper during the participant interview and perform the DBI calculations. After the visit, the pharmacist will transfer the data into the REDCap database, including a scanned version of the corresponding paper record. The study’s data manager will facilitate the review process by informing the senior pharmacist to review the DBI calculation and records. Upon successful review or correction of any errors, the record in REDCap will be locked.

After completing the trial, the data manager will export all data from REDCap, de-identify the records, check for missing values, outliers, and inconsistencies before passing the data to the study’s statistician for analysis. The analysis process will include further checks, such as range tests and outliers in computed and outcome values. Once the dataset’s quality is confirmed by the study’s data manager and statistician, the dataset will be locked.

To facilitate medication specific analyses, the dataset containing individual medication and medication use details will undergo post-processing to achieve consistency with medication names, dosage, units of measure and recording of “as needed” (PRN) medications. In addition, other relevant pharmaceutical data such as chemical compound identifiers or medication costs may be added to the dataset.

#### Outcome data

The dataset for analysis will include all interRAI assessments, admissions into residential care, hospital admissions, mortality, and the New Zealand Pharmaceutical Management Agency (PHARMAC) dispensing records. These will be classified into several groups as follows:

- Demographic data
- interRAI™ assessment data
- interRAI™ scales and Clinical Assessment Protocols (CAPs)
- New interRAI™-based frailty indices
- Drug Burden Indices (DBI)
- Medical and pharmaceutical notes that support the pharmacist deprescribing reviews
- Admissions into aged residential care
- New Zealand mortality data
- Dispensing records from PHARMAC
- Emergency department presentations and hospital admissions

Data from consented participants will be linked using each participant’s unique study ID. The final de-identified dataset will be provided to the study statistician for analysis. The dataset will be archived upon completion of the study.

#### Data monitoring and safety

The study is considered low risk. An internal data monitoring committee (DMC) independent from the funder and the investigators with no conflict of interest will be established to regularly monitor the study data integrity and quality as described above.

Any notification of unexpected events received by the study administrator will be recorded and passed to the data monitoring and safety committee for review. In addition, unexpected events and data trends requiring corrective action will be passed from the committee to the principal investigator (HJ) for follow-up.

#### Access to data

The study data manager will have full unrestricted access to all study data throughout the trial. The study administrator and study pharmacists will have access to personal details to facilitate recruitment, consenting, and booking visits to participants. No other member of the research team will have access to personal details of study prospects or study participants. The senior pharmacist will have restricted access to data as required to perform data review duties throughout the trial. As a member of the Data Monitoring Group, the study statistician will have access to raw data held in REDCap to perform data quality checks. Blinding will be maintained throughout the trial.

After completing the trial and locking of data, the primary investigator and other named members of the research team will have access to de-identified study data. Data will not be shared with other researchers as per ethics approval and participant consent.

### Outcomes

In this study, five outcome scenarios are possible: (1) completion of the trial, (2) entry into aged residential care (ARC) before completion (this would be linked to a change in GP and medication control), (3) prolonged hospitalisation, (4) death, and (5) withdrawal or other loss to follow-up. The first two outcome scenarios would involve the post medication review being undertaken either with the participant or via the medical records of a care facility. The third outcome scenario could lead to delays in recording the post-intervention review or may lead to any of the four other scenarios.

#### Primary outcome

The primary outcome will be the change in a participant’s DBI (ΔDBI) between the time of the baseline interRAI assessment (T1) and the time of 6 months of follow-up (T2; ΔDBI = DBI_T1_ – DBI_T2_). Data for the calculation will be collected by comparing medication use pre-and post-intervention. We will determine if there is a greater reduction in the DBI of participants in the experimental arm of the trial compared with participants with the same level of frailty in the control arm. Subgroup analysis will determine if deprescribing is more pronounced for those with more severe frailty.

#### Secondary outcomes

Secondary outcomes measures will be compared between the two arms of the trial after 6 months and are outlined in Table 3. From the funder’s perspective, these include the number of hospitalizations, emergency department visits, entry into aged residential care, all-cause mortality, and cost-utility. In addition, we will measure the number of emergency department visits and unplanned hospital admissions. Data on entry into or change in the level of care in aged residential care will be extracted from relevant national databases by the analytical services of the Ministry of Health. Patient mortality data will be matched using participants’ National Health Index (NHI) number, added to the dataset, and analyzed using competing risk regression. In a separate analysis, we will conduct a health cost utility assessment.

**Table 3:** Outcome measures and analysis

Outcome	Measure	Alternative hypothesis	Analysis
Drug burden change	Proportion ΔDBI ≥ 0.5	Intervention greater	*χ*^2^ comparison of proportions
Emergency department visits	Proportion	Remain the same or decrease	*χ*^2^ comparison of proportions
Unplanned hospital admissions	Proportion	Remain the same or decrease	*χ*^2^ comparison of proportions
Admissions into aged residential care	HR	Remain the same or decrease	CRRA
Mortality	HR	Remain the same or decrease	CRRA

*ΔDBI* change in Drug Burden Index, *WSR* Wilcoxon signed-rank test, *CRRA* competing risk regression analyses, *χ*^2^ chi-square

### Statistics methods

#### Power and sample size

For this RCT, we define the clinically significant change in DBI as 0.5, the equivalent of one medication contributing to DBI given at the minimal efficacious dose [14]. Approximately 4% of recent interRAI assessments show a ΔDBI ≥ 0.5 over 6 months. Therefore, a meaningful outcome from deprescribing in this study would be to increase the percentage, in the intervention cohort, with a DBI change ≥of 0.5 by 10% points. This would bring the percentage ΔDBI ≥ 0.5 over a 6-month period to 14%.

The null hypothesis is that there is no difference in proportions with a ΔDBI of ≥ 0.5 over 6 months between the control and intervention groups, given that 4% of participants in the control group have a ΔDBI of ≥0.5. Therefore, to disprove the null hypothesis, we need to detect a change in the number of participants of 10% or more with a ΔDBI of ≥0.5 with a power of 90% at an *α*=0.05. This requires 167 participants in each arm of the study, 334 in total.

For each frailty stratum under the null hypothesis, there will be no difference in change in DBI between the control and intervention groups and assume 4% of participants in the control group have a reduction in DBI of ≥0.5, then to detect a change in the number of participants of 20% or more with a reduction in DBI of ≥0.5 with a power of 80%, and at *α*=0.017 (0.05/3), requires 56 participants in each arm of the study (112 in each stratum, 336 in all).

It is estimated that over 12 months within the target areas of the district health boards’ approximately 650 participants will meet the basic inclusion criteria, including the minimum DBI. In addition, previous data suggest that approximately 50% of the study’s cohort will take the target medicines, and therefore, 325 people would be eligible to take part in the study per annum.

#### Statistical analysis

The data analysis will use the intention-to-treat principle (where all available data, including records showing non-adherence to the protocol, from participants will be included in the analysis). Thus, imputation will not be used for missing data.

We will compare the proportions of the outcome measures using a chi-squared test and present results with a 95% confidence interval.

We will present Kaplan-Meier survival curves for analysis of hospitalizations, emergency department visits, entry into aged residential care, and mortality within subgroups. Subgroups will include control low frailty, control medium frailty, control high frailty, intervention low frailty, intervention medium frailty, and intervention high frailty. The analyses will be controlled for age and sex, with death as a censored event. We will then conduct a competing risk analysis using cumulative incidence functions (CIF). For example, where entry to aged residential care is the primary event of interest, death is the competing event. The CIFs are the probability of observing these events before the end of the 6-month follow-up period.

#### Cost-benefit analysis

As a separate analysis, a health economist will oversee an analysis of financial costs of routine screening for frailty compared to the expected benefits from more appropriate prescribing, reduced pharmaceutical costs, and avoided hospital admissions and entry into residential care. Given that we will stratify our cohort by frailty, we anticipate identifying a group of participants with a degree of frailty who will benefit most from targeted medication reviews.

The cost-effectiveness analysis will include costs and benefits both for the participants and for the health care system. Standard robustness checks will be performed.

A desirable feature is that the benefits of implementing the intervention are likely to be realized soon after implementation, contributing to a favorable cost-effectiveness ratio [33].

#### Reporting and dissemination

The trial results will be disseminated to medical professionals and researchers via journal articles, to local health authorities and delivery organisations through presentations, newsletters and media interviews, and study participants who requested information on the study’s outcome via a simplified summary.

We will report according to the CONSORT reporting guidelines [34].

Authorship will be considered and granted using policies of the University of Otago [35] and respective journals. Funder and other contributors to the study or dissemination method will be acknowledged in the publication.

Disclosure of participant-level data is not consented to and will therefore not be made available.

### Blinding

Participants will be blinded to their study arm. The pharmacists conducting the first medication reviews will be blinded at the time of the medication review but made aware of allocation following the review when they open the envelopes at the first home visit. Pharmacists conducting post-intervention medication reviews will be blinded to the participants’ allocation in the follow-up home visits. Therefore, those pharmacists collecting medication data at either step will be blinded. Data collected for other outcome measures are collected independent of the study; thus, blinding is ensured. Patient unblinding is permitted in case of health concerns requiring immediate attention.

The study administrator and the data manager will have access to all participant data. The primary investigator and the study statistician will be blinded to participant data and allocation during the trial and unblinded following data verification and locking.

### Ethics and protocol changes

Ethics approval has been obtained from the Health and Disability Ethics Committee (HDEC) based on this study protocol in revision 8 under amendment AM06. Minor changes to consent forms and participant information sheet were made under amendments AM07–AM10. Changes to allow conducting the second medication review over the phone during COVID-19 lockdown were approved under amendment AM12. Any further changes made to protocol revision 8 will be communicated to the Health and Disability Ethics Committee, and approval will be sought to implement the amended protocol. Incremental changes to the Participant Information Sheet (PIS) and Consent to Contact form will be made under subsequent amendments.

Any protocol modifications will be internally reviewed by the DMC, by the funder HRC and, if required, approved by HDEC.

## Discussion

This protocol is for a pragmatic two-arm randomized controlled superiority trial to test deprescribing of anticholinergic and sedative medications in frail older patients living in a community setting. In addition, the study will determine if pharmacist-led medication reviews of DBI medications will lead to community GPs deprescribing DBI medications.

The trial design is unique in determining if a frailty measure will help identify older adults who would most benefit from deprescribing one or more of their medications.

If the trial findings show a reduction in participants’ DBI score, the intervention involving pharmacist-led medication reviews may be implemented to help increase the translation of deprescribing anticholinergic and sedative medications in clinical practice. Ultimately, deprescribing these medications has the potential to reduce the use of inappropriate medications and medication-related harm in older people living in the community, improving their overall health and wellbeing.

### Trial status

The trial is underway using protocol version 8 on December 20, 2018. Participant recruitment commenced on June 2, 2018, for the pilot phase and February 12, 2019, for the formal trial. Recruitment is expected to be completed by October 31, 2020, with data capture completed by May 31, 2021.

## Supplementary Information

**Additional file 1.** SPIRIT Checklist.

**Additional file 2.** General GP information letter.

**Additional file 3.** Invitation and Consent to Contact form.

**Additional file 4.** Patient Information Leaflet and Consent form.

**Additional file 5.** Medication review form.

**Additional file 6.** Frailty Index scoring sheet.

## Data Availability

Study materials are provided as supplementary materials. New Zealand ethics laws do not permit free sharing of data although aggregate and de-identified data may be provided to collaborating research groups under an appropriate data sharing agreement.

## Abbreviations

ANZCTR: Australian and New Zealand Clinical Trials Registry
ATC: Anatomical therapeutic chemical
CAP: Clinical Assessment Protocol
CDHB: Canterbury District Health Board
CIF: Cumulative Interference Function
CRRA: Competing Risk Regression Analyses
DBI: Drug Burden Index
DHB: District Health Board
GP: General practitioner
HDEC: Health and Disability Ethics Committees
HRC: Health Research Council of New Zealand
InterRAI-CA: InterRAI Contact Assessment
InterRAI-HC: InterRAI Home Care Assessment
NHI: National Health Index
SPIRIT: The Standard Protocol Items: Recommendations for Interventional trials
WSR: Wilcoxon signed-rank test
