# Supplementary material for: Deprescribing to reduce polypharmacy: study protocol for a randomised controlled trial assessing deprescribing of anticholinergic and sedative drugs in a cohort of frail older people living in the community
Source: Trials. 2021 Nov 3;22:766. doi: 10.1186/s13063-021-05711-w (PMC8564597; doi:10.1186/s13063-021-05711-w)
Supplement: Supplementary file 1 — Additional file 1. SPIRIT Checklist. [file 13063_2021_5711_MOESM1_ESM.docx]

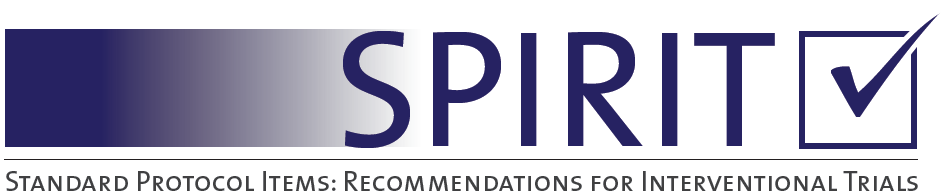


SPIRIT 2013 Checklist: Recommended items to address in a clinical trial protocol and related documents*

| Section/item | ItemNo | Description | Page |
| --- | --- | --- | --- |
| **Administrative information** | | |  |
| Title | 1 | Deprescribing to reduce polypharmacy: study protocol for a randomised controlled trial assessing deprescribing of anticholinergic and sedative drugs in a cohort of frail older people living in the community | 1 |
| Trial registration | 2a | Australian New Zealand Clinical Trials Registry: ACTRN12618000729224 | 3 |
|  | 2b | Australian New Zealand Clinical Trials Registry is a partner registry to WHO. | 3 |
| Protocol version | 3 | 20-12-2018 Version 8 | 1 |
| Funding | 4 | This work is funded via grant 17/363 of the Health Research Council, Level 3, 110 Stanley St, Grafton, Auckland 1010, New Zealand. | 19 |
| Roles and responsibilities | 5a | **Ulrich Bergler**, MSc, APSY, Department of Medicine, University of Otago, Christchurch, New Zealand; ulrich.bergler@otago.ac.nz  **Nagham Ailabouni**, PhD, UniSA Clinical & Health Sciences, University of South Australia, Adelaide, South Africa; Nagham.Ailabouni@unisa.edu.au  **John W. Pickering**, PhD, Professor, Department of Medicine, University of Otago, Christchurch, New Zealand; john.pickering@otago.ac.nz  **Sarah Hilmer**, PhD, Professor of Medicine, Medicine, Northern Clinical School, University of Sydney, Sydney Australia; sarah.hilmer@sydney.edu.au  **Dee Mangin**, PhD, Professor, University of Otago, Christchurch New Zealand; Professor and David Braley and Nancy Gordon Chair in Family Medicine, McMaster University, Canada; mangind@mcmaster.ca  **Prasad Nishtala**, PhD, Associate Professor, Department of Pharmacy & Pharmacology, University of Bath, England; P.Nishtala@bath.ac.uk  **Hamish Jamieson**, PhD, Senior Lecturer and Geriatrician, University of Otago Christchurch, Department of Medicine; Burwood Hospital, Christchurch, New Zealand; hamish.jamieson@otago.ac.nz  **Sponsor-investigator**: Health Research Council of New Zealand, Level 3/110 Stanley Street, Grafton, Auckland 1010, New Zealand  The study was conceived by HJ with input from SH, PSN, and DM; the protocol was developed by UB, NA, and with input from all authors; HJ leads the research team; JP conducts statistical analysis; UB is the data and project manager, which include all administration—ethics approval and reporting; SH, PSN, NA provide pharmaceutical expertise and review; DM provides guidance and support throughout all aspects of the study. All authors reviewed the study protocol and approved the final manuscript. | 1  19f |
|  | 5b | Dr Hamish Jamieson, University of Otago, Christchurch, Department of Medicine, 2 Riccarton Avenue, Christchurch Central, Christchurch 8011 |  |
|  | 5c | HRC will have no role in the design of the study, and its execution, analyses, interpretation of data, or decision to submit results. University of Otago Christchurch will be involved in participant recruitment and dissemination of study results. | 19 |
|  | 5d | Data will be housed in a secure database managed by UB at the research centre at the University of Otago, Christchurch. The steering committee consists of the authors of the protocol UB, NA, JWP, SH, DM, PH, HJ. | 11 |
| Introduction |  |  |  |
| Background and rationale | 6a | Background contains an evidence-based rationale for deprescribing anticholinergic and sedative medications in frail older adults. | 3-5 |
|  | 6b | This section also provides evidence for investigating the change in DBI medication use, hospitalisation rate, and entry into aged residential care rate after intervention. | 3-5 |
| Objectives | 7 | We hypothesise that implementing a pharmacist-led medication review and provision of deprescribing recommendations to general practitioners (GP) will reduce the use of anticholinergic and sedative medications in community-dwelling older people compared to the control arm overall and that the reduction will be more pronounced for older people with a greater level of frailty. Thus, the primary objective is to determine if pharmacist-led medication reviews focused on reducing anticholinergic and sedative medications lead to GPs deprescribing these medications for older people living in the community. The secondary objective is to determine if a frailty measure based on data collected in the interRAI Home Care(24) and Contact assessment (25) can identify a group of older people who could benefit the most from deprescribing. | 5 |
| Trial design | 8 | This will be a pragmatic two arm randomised controlled superiority trial to test general practitioner uptake of pharmacist recommendations to deprescribe anticholinergic and sedative medications that are causing adverse side effects in patients. Participants will be stratified into one of three frailty strata and allocated in equal numbers to the intervention and control arms of the trial. | 5-6 |
| Methods: Participants, interventions, and outcomes | | |  |
| Study setting | 9 | This trial will be conducted at the University of Otago, Christchurch, in the Canterbury provincial region of New Zealand. Study pharmacists will visit participants in their homes, where they will review medication use at the time of their visit. | 6 |
| Eligibility criteria | 10 | Participants will be eligible for inclusion if they  1) Are aged ≥65 years  2) Have undergone an interRAI Home Care or Contact assessment,  3) Are regularly taking at least one anticholinergic or sedative medication as shown in the interRAI or dispensing records  Participants will be excluded from the study for any of the following reasons:  1) Not consenting for their interRAI data to be used for research  2) Having a psychiatric disorder, or dementia disease (e.g. Alzheimer’s disease, dementia, schizophrenia, abnormal thought processes, delusions, hallucinations)  3) Scoring 3 or higher on the interRAI Cognitive Performance Scale  4) Having a terminal illness with life expectancy ≤6 months  5) Determining as non-frail by having no deficits in the frailty index  6) Having an initial DBI score of <0.5 (DBI score of 0.5 is the equivalent of one DBI medication being taken at the minimum efficacious dose)  7) Having a potentially life-threatening drug interaction requiring urgent medical intervention (during the study period) | 6-7 |
| Interventions | 11a | Using each consenting participant’s interRAI data, the study administrator will calculate his/her frailty index (FI) and classify him/her into one of the three frailty strata: low, medium, or high— prior to the home visit. Each frailty strata will have its own separate randomisation sequence. During the home visits, the study pharmacists will randomly allocate participants to either the study arm or intervention arm of the trial. This will be done by opening a sealed envelope from a participant’s frailty stratum to reveal if the participant will take part in the control or intervention arm of the study.  In the intervention arm, pharmacists will ask participants if they are experiencing any unwanted side effects from their DBI drugs. Using the outcomes of these discussions, pharmacists will recommend to GPs possible anticholinergic and sedative medications that their patients could benefit from having deprescribed.  In the control arm, during the home visit pharmacists discuss with participants their DBI medications, but do not recommend to GPs to deprescribe. In this regard, participants continue to receive standard care. | 8-10 |
|  | 11b | Care of all participants will be supervised by their GP. If during the trial a participant develops a potentially life-threatening drug interaction requiring urgent medical intervention S/he with be withdrawn from the study. | 10 |
|  | 11c | Participants in the intervention arm of the trial who consider that they are having adverse side effects from their DBI medications will visit their GPs for revision of their medications. | 10 |
|  | 11d | All clinical decision-making and care of participants will remain with their GP throughout the study. Study pharmacists will work in collaboration with GPs but will have no direct clinical responsibility. | 10 |
| Outcomes | 12 | In this study, five outcome scenarios are possible: 1) completion of the trial, 2) move to aged residential care prior to completion, 3) prolonged hospitalisation, 4) death, and 5) withdrawal or other loss to follow up.  The primary outcome will be the change in a participant’s DBI (ΔDBI) between the baseline interRAI assessment (T1) and 6 months follow-up assessment (T2; ΔDBI = DBIT1 – DBIT2). Data for the calculation will be collected by comparing BDI medication use pre- and post- intervention. We will determine if there is a greater reduction in the DBI of participants in the experimental arm of the trial compared with participants with the same level of frailty in the control arm. Subgroup analysis will determine if deprescribing is more pronounced for those who are frailer.  Secondary outcomes measures will be compared between the two arms of the trial after 6 months. These include number of hospitalisations, number entering aged residential care, number with all-cause mortality, and cost utility analysis from the funder’s perspective. We will also measure the number of emergency department visits and unplanned hospital admissions. | 13-14 |
| Participant timeline | 13 | For all participants’ medication reviews will be undertaken prior to randomisation and again at six months follow up. Data will be collected at the time of the interRAI assessment (T0), during preparation for the intervention (T1), at least 6 months following the baseline medication review (T2), and at the end of the study period, or end of study participation for participants admitted into aged residential care, or at death (T3). See Table 2. | 11  Table 2 |
| Sample size | 14 | For this RCT we define a clinically significant change in DBI to be 0.5, the equivalent of one medication contributing to DBI given at the minimal efficacious dose. Approximately 4% of recent interRAI assessments show a ΔDBI ≥ 0.5 over a six-month period. A meaningful outcome from deprescribing in this study would be to increase the percentage, in the intervention cohort, with a DBI change ≥0.5 by 10% points. This would bring the percentage ΔDBI ≥ 0.5 over a six-month period to 14%.  The null hypothesis is that there is no difference in proportions with a ΔDBI of ≥ 0.5 over a six-month period between the control and intervention groups, and assuming 4% of participants in the control group have a ΔDBI of ≥0.5. To disprove the null hypothesis we need to detect a change in number of participants of 10% or more with a ΔDBI of ≥0.5 with a power of 90% and at an α=0.05. This requires 167 participants in each arm of the study, 334 in total. For each frailty stratum under the null hypothesis there will be no difference in change in DBI between the control and intervention groups, and assume 4% of participants in the control group have a reduction in DBI of ≥0.5, then to detect a change in number of participants of 20% or more with a reduction in DBI of ≥0.5 with a power of 80%, and at α=0.017 (0.05/3), requires 56 participants in each arm of the study (112 in each strata; 336 in all).  It is estimated that over 12 months within the target areas of the district health boards’ approximately 650 participants will meet the basic inclusion criteria including the minimum DBI. Previous data suggests that approximately 50% of the study’s cohort will take the target medicines and therefore 325 people would be eligible to take part in the study per annum. | 14-15 |
| Recruitment | 15 | Potential participants will be contacted via two pathways: a) interRAI assessors will invite eligible older adults to participate in the study during their interRAI assessment home visit, and b) the local district health board will post letters to older adults who have recently had an interRAI assessment. The letters will outline a brief overview of the study with ‘Consent to Contact’ forms (CtC) and free post return envelopes.  Approximately 4% of recent interRAI assessments show a ΔDBI ≥ 0.5 over a six-month period. A meaningful outcome from deprescribing in this study would be to increase the percentage, in the intervention cohort, with a DBI change ≥0.5 by 10% points. This would bring the percentage ΔDBI ≥ 0.5 over a six-month period to 14%.  It is estimated that over 12 months within the target areas of the district health boards’ approximately 650 participants will meet the basic inclusion criteria including the minimum DBI. Previous data suggests that approximately 50% of the study’s cohort will take the target medicines and therefore 325 people would be eligible to take part in the study per annum.    Follow-up medication reviews by telephone were approved by the funder New Zealand Health Research Council and New Zealand Ethics and Disability Committee to ensure ongoing data collection during the New Zealand COVID-19 lockdown period from 23/3/2020 to 15/6/2020. | 7-8  Figure 1 |
| **Methods: Assignment of interventions (for controlled trials)** | | |  |
| Allocation: |  |  |  |
| Sequence generation | 16a | Predefined randomisation lists with a 1:1 allocation will be calculated by the study's data manager using a Mersenne Twister algorithm. The algorithm will be repeatedly run until a list is identified that shows a difference in the total number of intervention and control arm participants of not more than three participants at any given point in the process. Centralised allocation is considered neither viable nor required in this community-based study setting, as the participants become available at random through the interRAI assessment process. The randomisation list is securely stored on the University's computer system and not available to any other research team member. | 8 |
| Allocation concealment mechanism | 16b | Each frailty strata will have its randomisation list and set of treatment allocations concealed in sequentially numbered sealed opaque envelopes. Allocations will be made by the study administrator, who will select the next allocation matching a participant's frailty strata when the participant's home visit is booked. | 8 |
| Implementation | 16c | The allocation sequence will be generated by the data manager (co-author UB). Participants are enrolled by the study administrator in collaboration with the study pharmacists. During the home visits the study pharmacists allocate participants to either the intervention or control arm of the study according to a sequence of sealed opaque envelopes in their frailty strata. | 8 |
| Blinding (masking) | 17a | Participants will be blinded to their study arm. The pharmacists conducting the first medication reviews will be made aware of which arm of the trial participants are allocated to when they open the envelopes at the first home visit. In the follow-up home visits, different pharmacists conducting the post intervention medication review will be blinded to the participant’s allocation. | 16 |
|  | 17b | Participant unblinding is permitted in case of health concerns requiring immediate attention. | 17 |
| **Methods: Data collection, management, and analysis** | | |  |
| Data collection methods | 18a | For all participants’, their DBI medication use will be recorded prior to randomisation and again at six months follow up. Participants’ secondary health information such as mortality, hospital admissions, and fractures will be obtained from their health provider and the New Zealand Ministry of Health. These data will be linked with the study data using each participant’s unique national health index (NHI) number. | 11 |
|  | 18b | Participants may withdraw from the study at any time. | 8 |
| Data management | 19 | All observations and results will be recorded in custom designed Research Electronic Data Capture (REDCap https://www.project-redcap.org/) databases hosted at the University of Otago, Christchurch, New Zealand. The database will provide secure online data entry from multiple sites into a central data depository. | 11 |
| Statistical methods | 20a | The statistical analysis will use the intention-to-treat principle | 15 |
|  | 20b | We will present Kaplan-Meier survival curves for subgroup analysis. Subgroups will include control low frailty, control medium frailty, control high frailty, intervention low frailty, intervention medium frailty, intervention high frailty. These analyses will be controlled for age and sex, with death as a censored event. We will then conduct a competing risk analysis using cumulative incidence functions (CIF). For example, for entry into residential care, entry is the primary event of interest and death is the competing event and the CIFs are the probability of observing these events before the end of the 6-month follow-up period. | 15 |
|  | 20c | Records showing non-adherence to the protocol or missing data will be included in the intention to treat analysis. Imputation will not be used for missing data. | 15 |
| **Methods: Monitoring** | | |  |
| Data monitoring | 21a | An internal data monitoring committee (DMC) independent from the sponsor (HJ) and the investigators with no conflict of interest has been established to regularly monitor the study data integrity and quality. | 12-13 |
|  | 21b | Any notification of unexpected events received by the study administrator will be recorded and passed to the data monitoring and safety committee for review. Unexpected events, trends in data that require corrective action will be passed from the committee to the principal investigator (HJ) for follow up. The data manager (UB), project statistician (JWP) and DMC will have access to preliminary data. | 12-13 |
| Harms | 22 | Participants will be supervised by their GPs, who will manage any adverse events from deprescribing. Participants who suffer an adverse event will be withdrawn from the trial. | 10 |
| Auditing | 23 | The trial will be audited by regular reporting to the funder—Health Research Council of New Zealand. | N/A |
| Ethics and dissemination | | |  |
| Research ethics approval | 24 | Ethics approval (17CEN265) was granted by the Human Disability and Ethics Committee of New Zealand on 19 February 2019. Various amendments were proved by the ethics committee as follows:  1. Amendment 7 was processed to add an optional consent for the research team to contact community pharmacists for medication data, documented by:  • PAF_Form AM07 - minor changes to Consent to Contact form  • HDEC Letter 17CEN265AM07_Approved Amendment  2. Ethics amendment 8 was processed to improve the Patient Information Sheet and adding the National Health Index number to the consent form, documented by:  • PAF_Form AM08 - Update to the Person Information Sheet and Consent form  • HDEC Letter 17CEN265AM08_Approved Amendment  • Patient Information Leaflet and consent form (current version 20190220a)  3. Ethics amendment 9 request involved adding the option for the research team to access interRAI screening data on consent to contact form, documented by:  • PAF_Form AM09 - request to add consent to access interRAI screening data  • HDEC_Letter_17CEN265AM09_Approved_Amendment_pdf  4. Ethics amendment 10 requested to accept participant’s verbal consent to access screening data, documented by:  • PAF_Form AM10 - request for approval of verbal consent3   - HDEC_Letter_17CEN265AM10_Provisional_Approval_Amendment   • Response re Ethics ref 17CEN265AM10  • HDEC Letter_17CEN265AM10_Amendment_ApprovedNSC  • Invitation and Consent to Contact form 20190925 | 17 |
| Protocol amendments | 25 | While we had 8 protocol modifications agreed by all investigators and the trial funder and ethics committee, version 8 of the protocol has not changed since recruiting commenced.  1. Version 8 was approved by the funder HRC under variation 1539 and by HDEC under 17CEN265 amendments 5 and 6 evidenced by the below corresponding documents:  • 15395-JamiesonH-HRC Variation Request-20181030  • 15395-JamiesonH-HRC Variation Request signed  • HDEC_Letter_17CEN265AM05_Amendment  • PAF_Form AM06 - protocol corrections and add consent to contact community pharmacy  • HDEC Letter_17CEN265AM06_Approved_Amendment  2. A further and final amendment to the study protocol was processed as a deviation to the procedure during COVID-19 lock-down. To avoid an untenable delay in the trial during lockdown, we obtained both funder and ethics approval to continue follow up medication reviews by phone interview. I attach the document relating to this:  • PAF_Form AM12 - De-prescribing protocol variation due to covid-19 lockdown  • HDEC Letter 17CEN265AM12_Approved Amendment_COVID19  3. Several letters of support serve as evidence for review and acceptance of the protocol.  • Dee Mangin letter of support to HRC  • Letter of Support to HRC by Carolyn Gullery CDHB  • Phil Wood letter to HRC  Note: amendment AM11 is the identifier for the progress report to the Ethics committee  4. A further and final amendment to the study protocol was required during COVID-19 lock-down. To avoid an untenable delay in the trial during lockdown, we obtained both funder and ethics approval to continue follow up medication reviews by phone interview. I attach the document relating to this:  • PAF_Form AM12 - De-prescribing protocol variation due to covid-19 lockdown  • HDEC Letter 17CEN265AM12_Approved Amendment_COVID19 | 17 |
| Consent or assent | 26a | Informed consent will be obtained from potential participants using several pathways and steps. Firstly, interRAI assessors will invite eligible older adults to participate when they are assessing them at home. Secondly, the local district health board will post letters of invitation to older adults who have recently had an interRAI assessment but were not asked to participate in the trial. Once participants have expressed interest in participating in the study, the study administrator will contact them explaining the details of the trial. Final consent will be obtained by the study pharmacists during the baseline home visit. | 7-8 |
|  | 26b | Participants will give consent for the study team to contact their community pharmacists to obtain their prescribing records during the study period. | Consent form |
| Confidentiality | 27 | Data on study prospects (identified) is held in a dedicated secure ‘prospects’ database, while deidentifed participant data will be stored in a secure ‘study’ database using unique identification numbers. | 11 and 13 |
| Declaration of interests | 28 | There are no competing interests for the PI or any of the study team. | 19 |
| Access to data | 29 | The study data manager will have full unrestricted access to all study data throughout the trial. The study administrator and study pharmacists will have access to personal details to facilitate recruitment, consenting and booking of visits to participants. No other member of the research team will have access to personal details of study prospects or study participants. The senior pharmacist will have restricted access to data as required to perform data review duties throughout the trial. The study statistician as a member of the Data Monitoring Group will have access to raw data held in REDCap for the purpose of performing data quality checks.  Following completion of the trial and locking of data the Primary investigator and other named members of the research team will have access to de-identified study data. Data will not be shared with other researchers as per ethics approval and participant consent. | 13 |
| Ancillary and post-trial care | 30 | All participants will remain under the care of the GP before, during, and after the trial. | 10 |
| Dissemination policy | 31a | The outcome of the trial will be disseminated via journal article publication, newsletters, television and radio interviews, and verbal presentations to colleagues and community groups.  Study participants may request a simplified summary  The study protocol itself is published via a journal article | 16  19 |
|  | 31b | Authorship will be considered and granted using policies of the University of Otago and respective journals. Funder and other contributors will be acknowledged.  All authors of the protocol meet the eligibility guidelines. A professional writer, Dr Joanne Deely, contributed to preparing the HRC grant application, and preparing this protocol for publication. | 16  19-20 |
|  | 31c | Disclosure of the of the full dataset outside of the study team is not consented as per ethics approval. Any further information can be obtained by contacting the PI Dr Hamish Jamieson at: Hamish.jamieson@otago.ac.nz. | 16 |
| Appendices |  |  |  |
| Informed consent materials | 32 | The appendix includes the Consent to Contact from, the Participant Information sheet, and the Consent form |  |
| Biological specimens | 33 | No biological specimens were used in this study. |  |

DMC=data monitoring committee, IRB= institutional review board, REC=research ethics committee; SPIRIT=Standard protocol items: Recommendations for Interventional trials
