# Supplementary material for: Deprescribing to reduce polypharmacy: study protocol for a randomised controlled trial assessing deprescribing of anticholinergic and sedative drugs in a cohort of frail older people living in the community
Source: Trials. 2021 Nov 3;22:766. doi: 10.1186/s13063-021-05711-w (PMC8564597; doi:10.1186/s13063-021-05711-w)
Supplement: Supplementary file 2 — Additional file 2. General GP information letter. [file 13063_2021_5711_MOESM2_ESM.pdf]

Canterbury District Health Board
Te Poari Hauora ō Waitaha

UNIVERSITY of OTAGO
Te Whare Wānanga o Otāgo
NEW ZEALAND

South Canterbury District Health Board

Deprescribing as an intervention to Polypharmacy:
a protocol for a Randomised Controlled Trial in older community people.

16 October 2018

To Whom It May Concern:

I am writing to inform you of a study that is currently being conducted by the University of Otago in collaboration with the Canterbury and the South Canterbury District Health Boards.

This study aims to reduce polypharmacy and patients' medication burden by deprescribing. Deprescribing is the process of safely reducing or discontinuing medications that are deemed to be inappropriate or no longer necessary. This study will involve targeted medication review of older community dwelling adults who have recently undergone a needs assessment using the interRAI Homecare assessment tool. Targeted are anticholinergic and sedative medications, as these medications are commonly prescribed in older people and are associated with many adverse effects including poor cognitive and physical functioning. Deprescribing has been associated with a lower annual acute hospital admission rate, an improvement in quality of life.

If one or more of your patients has consented to participate in the study, a pharmacist will visit them at their home. After reviewing the participant's medication regimen, drug-specific deprescribing protocols will be utilised to put forward suggestions to you as the participants' general practitioner of drugs that may be suitable to deprescribe (i.e. reduce or discontinue). The details of these recommendations will be summarised in a deprescribing medication review report and this will be mailed to you. We want to emphasise that the pharmacist will not initiate changes and all clinical care and decision making remains with you as the General Practitioner. Testing this method and its effects may benefit you and your patients. It may also provide benefit to others if it proves feasible and successful.

We have registered this randomised controlled study in the Australian New Zealand Clinical Trials Registry ACTRN12618000729224p. Ethical approval for this study has been obtained from the Human & Disability Ethics Committee (HDEC) with reference 17/CEN/265. Feel free to contact me in regards to the study or request a copy of the study protocol.

Yours sincerely,

Dr Hamish Jamieson – Geriatrician
Contact phone number: 0800 ageing / 0800 243 464
Contact email address: better.ageing@otago.ac.nz
