# Supplementary material for: Deprescribing to reduce polypharmacy: study protocol for a randomised controlled trial assessing deprescribing of anticholinergic and sedative drugs in a cohort of frail older people living in the community
Source: Trials. 2021 Nov 3;22:766. doi: 10.1186/s13063-021-05711-w (PMC8564597; doi:10.1186/s13063-021-05711-w)
Supplement: Supplementary file 3 — Additional file 3. Invitation and Consent to Contact form. [file 13063_2021_5711_MOESM3_ESM.pdf]

Invitation to find out more about an important healthcare study.

We would like to invite you to participate in a research study on how to best reduce the adverse side effects of polypharmacy in the elderly. The study is conducted by the University of Otago in collaboration with the Canterbury and South Canterbury district health boards.

Many senior citizens take multiple medications every day to improve their health. From time to time, it is sensible to review all medications a person is taking to ensure the best possible health outcome is achieved. This research study aims to establish if a pharmacist-led medication review will support your General Practitioner in achieving the best health outcome for you, and other older people.

We want to hear from you if you are interested to learn more about the study and potentially take part. Be assured that your personal well-being is central to this study and contacting us will not commit you in any way to take part. If you want to know more and have a conversation about what would be involved, then please return the attached form to us giving permission to contact you. For the study team to assess if you are meeting the criteria for the study, we are asking you to also give permission to access the result of your recent interRAI™ needs assessment and to contact your pharmacy to check if you are taking any of the medications considered in this study.

Our study administrator will then be in contact with you.

Thank you for reading and considering this invitation.

Study Administrator

Phone: 0800 243 464 (0800 AGEING)

Text: 021 279 1757

Email: better.ageing@otago.ac.nz

Consent to be contacted

Declaration by participant:

I have read the invitation letter and would like to be contacted to learn more about this study.

My name:

My phone number:

Days and times best suited are:

Other details:

☐ **I permit** my recent interRAI™ assessment data to be accessed by the study team to check if I am eligible to take part in the study.

☐ **I permit** my pharmacist to provide information to the study team if I am regularly taking any of the medications considered by the study.

My Pharmacy:

Please hand the signed form to the assessor or post it to us using the attached prepaid envelope. Thank you

Signature: **Date:**

**Administrative Use Only**	
☐ CDHB ☐ SCDHB ☐ Access Community Health ☐ Healthcare NZ ☐ Nurse Maude	
Assessor:	The patient is taking:<br>☐ Sleeping pills / Anxiety meds / Antidepressants<br>☐ Pain relief, other than Paracetamol<br>☐ Urinary incontinence medication
NHI :	
