# Supplementary material for: Deprescribing to reduce polypharmacy: study protocol for a randomised controlled trial assessing deprescribing of anticholinergic and sedative drugs in a cohort of frail older people living in the community
Source: Trials. 2021 Nov 3;22:766. doi: 10.1186/s13063-021-05711-w (PMC8564597; doi:10.1186/s13063-021-05711-w)
Supplement: Supplementary file 4 — Additional file 4. Patient Information Leaflet and Consent form. [file 13063_2021_5711_MOESM4_ESM.pdf]

Participant Information Sheet

Study title:	**Deprescribing as Intervention to Polypharmacy: A Randomised Controlled Trial (RCT) in older community patients**
Ethics approval:	17/CEN/265
Locality:	Canterbury and South Canterbury DHB, New Zealand
Lead researcher:	Dr. Hamish Jamieson
Study phone number:	0800 AGEING (0800 243 464)

You are invited to take part in a study about deprescribing. Deprescribing is the process of reducing and/or discontinuing medicines that may be inappropriate, harmful or no longer necessary. Whether or not you take part is your choice. If you do not wish to take part, you don't have to give a reason, and it won't affect the care you receive. If you wish to take part now, but change your mind later, you can withdraw from the study at any time.

This participant information sheet will help you decide if you wish to take part. Feel free to discuss the study with other people, such as family, whānau, friends, or healthcare providers.

Why are we doing the study?

Older patients are sometimes prescribed medicines they no longer need. The more medicines you take, the more susceptible you are to experiencing one or more negative health effects. In particular, sedative and anticholinergic medicines tend to be overprescribed to older people. Side effects such as confusion, dizziness, poor quality of sleep and an increased number of falls have been reported with use of these medicines.

Why are we inviting you to take part in this study?

You are or have recently been assessed by a District Health Board assessor using the standardised interRAI™ tool which showed that you meet the selection criteria for this important study.

What would your participation involve?

This study aims to investigate whether it is feasible to reduce or discontinue these medicines and whether this will improve quality of life and wellbeing. This study is designed as a randomized control trial, meaning that as a participant you are randomly placed in either the intervention or the control group. Participants in both groups will continue to receive normal clinical care through their GP.

Your decision to take part in the study or not has no impact on the outcome and options available to you as a result of the assessment.

Once we receive your signed consent form, an administrator from your District Health Board will contact you to arrange a time and location convenient for you to meet with the pharmacist.

At this appointment, the pharmacist will record all the medications you take and may have an in-depth discussion with you about your current experience and any concerns you may have about any of your prescribed medicines.

The purpose of this discussion is to ascertain any medicine(s) that are sedative or anticholinergic that could be causing you problems. The appointment will involve approximately 30-60 minutes of your time. You may wish to invite any of your relatives, family or whānau to this appointment.

What happens after the initial appointment?

The pharmacist may write a recommendation in regards to some of your medications for discussion with your GP, and send this to your GP. During your next GP visit your regular GP may discuss this recommendation with you and together you will decide on any changes to your current medications.

IMPORTANT: Do not make changes to your medication before you have discussed those with your GP! It is <u>not</u> urgent that you see your GP.

After about 6 months a pharmacist or nurse will visit you again and record any changes in your medications. This will take approximately 10-20 minutes of your time.

What are the possible benefits and risks to you of participating?

If you decide to participate in this study, you may experience one or more possible health benefits. You may feel better overall as you will have a reduced risk of suffering from undesirable side-effects of your medicines. You may also feel more mobile and active.

On the other hand, you may not experience any benefits from stopping any of your medicines. When reducing or stopping anticholinergic and sedative medicines), some patients may be susceptible to developing adverse drug withdrawal effects (ADWEs). ADWEs occur because your body may have become used to the medicines after being prescribed them for a long period of time. To prevent and reduce your risk of developing ADWEs, all target medicines will be slowly reduced or discontinued.

If you experience any unexpected adverse effect, or if you are not feeling well, you should contact your GP for medical attention as usual. Your GP will ascertain the reason to why you have been feeling unwell and establish if they are the likely result of reducing or discontinuing your medicines, or due to other medical conditions.

You may choose to withdraw from the study at any time with no disadvantage to yourself.

What would happen if you were injured in the study?

If you were injured in this study, which is unlikely, you would be eligible to apply for compensation from ACC just as if you were injured in an accident at work or at home.

This does not mean your claim will automatically be accepted. You will have to lodge a claim with ACC, which may take some time to assess. If your claim is accepted, you will receive funding to assist in your recovery. If you have private health or life insurance, you may wish to check with your insurer that taking part in this study won't affect your cover.

What are your rights as a participants in the study?

If you decide to participate in the study, your records will be held with reference to your National Health Index number without your personal name or contact details being included in the information that will be collected. All of the collected health information will be securely stored using a database designed for medical research at the University of Otago and future health data will be collected from several systems for holding relevant data to follow your progress. Members of the research team (listed on page 5) may access this information, during the course of the study.

Participation in this study is voluntary and it is completely up to you whether you agree to participate or decline. You are also free to seek advice from your GP, family member(s), relatives or friends about participating in this study. If you decide to participate in this study, you have the right to withdraw from the study and decline continuing to participate at any stage. You do not have to provide a full reason for why you do not wish to continue. However, this information would be very helpful and useful to the study.

You have the right to request your health information collected through this study to be deleted or altered. We will amend your health information records according to your feedback, as appropriate.

No payment or reimbursement will be provided for participating in this study.

What will happen after the study ends, or if you pull out?

No further medical intervention will occur after the conclusion of the study. Future health information will be obtained and securely stored in such a way that only those researchers mentioned below will be able to gain access to it.

At the end of the project, any personal information will be destroyed immediately except that any raw data on which the results depend will be retained in secure storage for ten years after which it will be destroyed.

Any reports about this project will contain information that is amalgamated for all the participants as a group, so it will not be possible to identify any individual in any of these reports. You are welcome to request a copy of the results of the project from the researchers.

The results of the project may be published in peer-reviewed scientific journals. This may occur one to two years after the completion of the study. The publication will be emailed to the GPs who have been involved in conducting the study. You may request for it to be emailed to yourself.

More information about the study

This study is funded by an independent organization, the Health Research Council. Ethics approval to carry out this study has been granted by the Health and Disability Ethics committee.

The study is being carried out by the following researchers:

- Dr Hamish Jamieson, Senior Lecturer and Geriatrician,
  University of Otago and Canterbury District Health Board
- Dr Prasad Nishtala, Senior Lecturer,
  School of Pharmacy, University of Otago
- Professor Dee Mangin, David Braley Chair in Family Medicine
  Professor and Associate Chair (Research), McMaster University
- Ulrich Bergler, Research Fellow and Project Manager,
  University of Otago and Canterbury District Health Board
- Rebecca Abey-Nesbit, PhD Candidate & Researcher-Analyst,
  University of Otago and Canterbury District Health Board
- Associate Professor John Pickering, Department of Medicine,
  University of Otago

Contact information

This information sheet is for you to keep.

If you have any question or concerns about the study, you may contact the study administrator or request to be contacted by any of the researchers involved:

Phone: 0800 AGEING (0800 243 464)

Email: better.ageing@otago.ac.nz

If you want to talk to someone who isn't involved with the study, you may contact:

Independent health and disability advocate
Phone: 0800 555 050 or email advocacy@hdc.org.nz .

Independent Māori Support for the study
Irihapeti Bullmore; Kaumatua Clinical Assessor; Phone: 0274 677 573

If you are concerned about the way this study is being conducted or you wish to make a formal complaint, please contact the
Health and Disability Ethics Committee (HDEC) that approved this study.
Phone: 0800 438 442 orEmail:hdecs@moh.govt.nz

Participant Consent Form

Declaration by participant:

I have read, or have had read to me in my first language, and I understand the Participant Information Sheet.

I have had the opportunity to ask questions and I am satisfied with the answers I have received.

I agree to participate in this study and give permission for the researchers to access and use all my medication and medical data, including data held by the Ministry of Health, PHARMAC or in computerised systems such as Momentum, HealthOne and Health Connect South, for this research project.

I have been given a copy of the Participant Information Sheet and Consent Form to keep.

Participant's name: **NHI:**

Signature: **Date:**

Declaration by the interRAI™ assessor or Pharmacist:

I have given a verbal explanation of the research project to the participant, and have answered the participant's questions about it.

I believe that the participant understands the study and has given informed consent to participate.

Assessor / Pharmacist name:

Signature: Date:
