# Supplementary material for: Deprescribing to reduce polypharmacy: study protocol for a randomised controlled trial assessing deprescribing of anticholinergic and sedative drugs in a cohort of frail older people living in the community
Source: Trials. 2021 Nov 3;22:766. doi: 10.1186/s13063-021-05711-w (PMC8564597; doi:10.1186/s13063-021-05711-w)
Supplement: Supplementary file 5 — Additional file 5. Medication review form. [file 13063_2021_5711_MOESM5_ESM.pdf]

Name :

NHI:

DOB :

Date:

Study No:

Reconciliation: Pre / Post

Performed: Visit / Phone

Next GP visit:

Adherence: 1 2 3 4 5

Regular meds

Disp Containers / Blisterpacks / Self-filled

Medication	Strength	Frequency	Notes	Eligible med	DBI

PRN and OTC meds

Medication	Strength	Frequency	Est. actual daily dose in last week	Eligible med	DBI

Please note PRN calculations on the back side of this form

Total DBI :
