# Supplementary material for: Deprescribing to reduce polypharmacy: study protocol for a randomised controlled trial assessing deprescribing of anticholinergic and sedative drugs in a cohort of frail older people living in the community
Source: Trials. 2021 Nov 3;22:766. doi: 10.1186/s13063-021-05711-w (PMC8564597; doi:10.1186/s13063-021-05711-w)
Supplement: Supplementary file 6 — Additional file 6. Frailty Index scoring sheet. [file 13063_2021_5711_MOESM6_ESM.pdf]

FRAILTY INDEX SCORING SHEET

Pharmacist | **date** | **time**

Name | **DOB** | **NHI**

☐ **CA** / ☐ **HC** | **Assessment date** | ☐ **Consent to access Momentum**

CA/HC	Question	Value labels	score	actual
C1/C1	Cognitive skills for daily decision making	0 Independent	0	
		1 Modified independence<br>2 Minimally impaired<br>3 Moderately impaired<br>4 Severely impaired<br>5 No discernible consciousness, coma	1	
C2a/G2a	Bathing	0 Independent<br>1 Independent, setup help only	0	
		2 Supervision- oversight, cueing<br>3 Limited assistance- guided manoeuvring of limbs<br>4 Extensive assistance- 1 person weight bearing support<br>5 Maximal assistance- 2 person weight bearing support<br>6 Total dependence<br>8 Activity did not occur	1	
C2b/G2b	Personal hygiene	0 Independent<br>1 Independent, setup help only	0	
		2 Supervision- oversight, cueing<br>3 Limited assistance- guided manoeuvring of limbs<br>4 Extensive assistance- 1 person weight bearing support<br>5 Maximal assistance- 2 person weight bearing support<br>6 Total dependence<br>8 Activity did not occur	1	
C2c/G2c	Dressing lower body	0 Independent<br>1 Independent, setup help only	0	
		2 Supervision- oversight, cueing<br>3 Limited assistance- guided manoeuvring of limbs<br>4 Extensive assistance- 1 person weight bearing support<br>5 Maximal assistance- 2 person weight bearing support<br>6 Total dependence<br>8 Activity did not occur	1	
C3/J3	Dyspnoea	0 Absence of symptom	0	
		1 Absent at rest, but present with moderate activity<br>2 Absent at rest, but present with normal activity<br>3 Present at rest	1	
C4/J7	Self-Reported Health	0 Excellent<br>1 Good	0	
		2 Fair	0.5	
		3 Poor<br>8 Could not, would not respond	1	
D2/D2	Ability to understand others	0 Understands	0	
		1 Usually understands<br>2 Often understands	0.5	
		3 Sometimes understands<br>4 Rarely or never understands	1	
D3/E2c	Self-reported mood- Sad	0 Not in the last 3 days	0	
		1 Not in the last 3 days, but often feels that way<br>2 In 1-2 of last 3 days<br>3 Daily in last 3 days<br>8 Person could not, would not respond	1	
			**Subtotal**	

			Subtotal	
D7/J1a, b	Falls	0 No fall in last 90 days	0	
		1 No fall in last 30 days but fell 31-90 days ago<br>2 One fall in last 30 days<br>3 Two or more falls in last 30 days	1	
D8a/J2c	Dizziness	0 Not present	0	
		1 Present but not exhibited last 3 days<br>2 Exhibited on 1 of last 3 days<br>3 Exhibited on 2 of last 3 days<br>4 Exhibited daily in last 3 days	1	
D9a/J5a	Pain frequency	0 No pain	0	
		1 Present but not exhibited in last 3 days	0.5	
		2 Exhibited on 1-2 of last 3 days	0.75	
		3. Exhibited daily in last 3 days	1	
D9b/J5b	Pain intensity	0 No pain	0	
		1 Mild	0.5	
		2 Moderate	0.75	
		3 Severe<br>4 Pain horrible/excruciating	1	
D11a/K2e	Decrease in amount of food or fluid usually consumed	0 No	0	
		1 Yes	1	
D11b/K2a	Weight loss of 5% or more in last 30 days, 10% or more in last 180 days	0 No	0	
		1 Yes	1	
D13/L1-4	Skin problem<br>If any iL1 to iL4>0	0 No	0	
		1 yes	1	
			**TOTAL**	
			**missing**	
			**FI**	
		0 < LOW ≤ 0.2 < MEDIUM ≤ 0.35 < HIGH ≤ 1	**Stata**	
